# A smartphone-based online system for fall detection with alert notifications and contextual information of real-life falls

**DOI:** 10.1186/s12984-021-00918-z

**Published:** 2021-08-10

**Authors:** Yaar Harari, Nicholas Shawen, Chaithanya K. Mummidisetty, Mark V. Albert, Konrad P. Kording, Arun Jayaraman

**Affiliations:** 1 Max Nader Rehabilitation Technologies and Outcomes Lab, Shirley Ryan Ability Lab, IL Chicago, USA; 2 Department of Physical Medicine and Rehabilitation, Northwestern University, Chicago, IL USA; 3 Medical Scientist Training Program, Northwestern University Feinberg School of Medicine, Chicago, IL USA; 4 Department of Computer Science and Engineering, University of North Texas, Denton, TX USA; 5 Departments of Bioengineering and Neuroscience, University of Pennsylvania, Philadelphia, PA USA

**Keywords:** SmartPhone, Fall detection system, Real-falls

## Abstract

**Background:**

Falls are a leading cause of accidental deaths and injuries worldwide. The risk of falling is especially high for individuals suffering from balance impairments. Retrospective surveys and studies of simulated falling in lab conditions are frequently used and are informative, but prospective information about real-life falls remains sparse. Such data are essential to address fall risks and develop fall detection and alert systems. Here we present the results of a prospective study investigating a proof-of-concept, smartphone-based, online system for fall detection and notification.

**Methods:**

The system uses the smartphone’s accelerometer and gyroscope to monitor the participants’ motion, and falls are detected using a regularized logistic regression. Data on falls and near-fall events (i.e., stumbles) is stored in a cloud server and fall-related variables are logged onto a web portal developed for data exploration, including the event time and weather, fall probability, and the faller’s location and activity before the fall.

**Results:**

In total, 23 individuals with an elevated risk of falling carried the phones for 2070 days in which the model classified 14,904,000 events. The system detected 27 of the 37 falls that occurred (sensitivity = 73.0 %) and resulted in one false alarm every 46 days (specificity > 99.9 %, precision = 37.5 %). 42.2 % of the events falsely classified as falls were validated as stumbles.

**Conclusions:**

The system’s performance shows the potential of using smartphones for fall detection and notification in real-life. Apart from functioning as a practical fall monitoring instrument, this system may serve as a valuable research tool, enable future studies to scale their ability to capture fall-related data, and help researchers and clinicians to investigate real-falls.

## Introduction

Falls are the second leading cause of accidental death worldwide, annually resulting in 646,000 mortalities and 37.3 million injuries that are severe enough to require medical attention [1]. Falls also constitute an economic burden, resulting in annual medical costs of approximately $50 billion in the US alone [2]. The risk of falling is especially high in populations that suffer from balance impairments such as older adults, amputees, or individuals with neurological disorders [3–6]. In many cases, fallers remain physically inactive for long periods, which can result in other secondary complications [7–10]. Fallers often develop a fear of falling which can lead to limited functional mobility resulting in sedentary lifestyles, depression, institutionalization, and indirectly contribute to elevated mortality [11]. The ability to detect and report a fall in real-time might also help to reduce the response time of EMS providers.

A well-developed fall detection system would provide increased understanding of falls in real life and their causes. The majority of falls occur without clinical supervision. In multiple cases, fewer than 20 % of falls were documented by medical professionals [12, 13], which limits our understanding of what precipitated these falls. Additionally, collecting data from fall events directly reduces the need for retrospective studies which are biased by a limited recall of subjects, even in the case that interviews are conducted frequently [14]. Finally, a system providing real-time monitoring of falls and near-falls could lead to emergency alerts for immediate medical attention, as well as provide data for post-hoc systematic analysis to develop improved, personalized fall prevention strategies. Improving and evaluating fall detection models is thus an important topic for research.

To develop and validate a fall detection model requires an extensive database, including recordings of many real-life falls and fall-like events. Real-life falls are rare events, and approximately 1400 recording days may be required to capture just one fall even in individuals with an elevated risk of falling [15, 16]. Given the infrequency of real-life falls during unstructured recording periods, most fall detection studies tend to use simulated falls performed in laboratory settings [17–20]. However, there are characteristics of real-life falls that are not present in lab-acquired falls, which could impact the quality of detection methods [21]. Recently, a consortium of research collaborators has assembled a set of real-world falls by collecting acquired fall data (FARSEEING consortium [22]), and several studies have used this repository to develop and validate fall detection models [15, 23]. However, many of these follow-up studies are limited by the type of data collected, which presents difficulties for the proper validation on unique populations. Developing better methods of producing diverse, high quality real-life falls and near-falls data could thus be of very high importance.

An often overlooked but critical aspect of validating fall detection models with a limited set of data is an appropriate choice of negative test cases (i.e., non-fall events). Many studies contrast fall detection with standard activities of daily living (ADLs) [23–26]. However, this can lead to inflated accuracies that do not generalize well. Alternatives include sets of natural behaviors that have a high probability of being incorrectly identified as falls, such as the high-impact non-fall events [27]. These non-fall or fall-like incidents, such as stumbles, can contain information which is valuable for clinical evaluation of fall probability, [28]. By recording high-impact, non-fall events, a more challenging retrospective analyses can be performed on future developed models.

Only a small number of prospective studies deployed fall detection systems into the community and validated their performance in real-time [29]. The majority of systems that tested their fall detection systems in real life have used inertial sensors, commonly accelerometers with or without gyroscopes, for detecting the falls [30–36]. However, all of these systems currently require the use of dedicated sensors which are externally attached to the users’ bodies. Using these sensors required technical intervention and assistance to attach or remove the sensors [30], charge or replace the sensor [33], or download the data [31], making the process less feasible and non-user-friendly. Additionally, these fall systems are only effective when the device is being utilized and incorporated into the user’s daily life. Therefore, there is a need for a fall detection system that uses sensors embedded in an everyday accessory that the participants already use in their daily living, such as their smartphone [23, 37–39]. While smartphone devices have been explored in the previously published prospective studies, a major drawback is that all of them except one [35] were offline and could not send a real-time fall notification. Therefore, there is a need for an online system which is capable of sending a timely alert of a fall to a researcher, relevant caregiver, or emergency medical services. However, variability in fall-detection performance may occur across different hardware models, operating systems and device usage levels. Although device-specific performance was not explored in the current study (the device model and operating system version were kept fixed), it remains an important consideration before implementing this approach on user’s personal devices.

Machine learning algorithms are increasingly used in the development of fall detection models [17], yet currently all fall-detection machine-learning studies but one [30] are retrospective. Development and validation of machine learning models in retrospective introduces a risk of overfitting (leakage), where algorithms are effectively using their developers’ knowledge of the test set. This may result in systems with lower-than-expected performance on new datasets. Testing the performance of machine learning models in prospective studies is crucial in order to understand their true ability to detect real-life falls.

Here we present a prospective study investigating the performance of a smartphone-based, online, real-life fall detection system. The system developed in this study includes a machine-learning classifier for detecting falls based on the smartphone’s accelerometer and gyroscope data. The system also includes an activity recognition model, a location tracker, a web portal for data exploration, and a real-time online notification system, which generates a timely notification of the fall and the faller status.

## Materials and methods

### Study design

We developed a smartphone-based system for real-time fall detection and alert generation. The system’s performance was investigated in a prospective study for 23 individuals with varied diagnoses and conditions, leading to an elevated risk of falling. All the participants signed informed consent before study participation, which was approved by the Northwestern University institutional review board (NUIRB, IL, USA). All the study procedures were carried out in accordance with the standards listed in the Declaration of Helsinki 1964.

All consented participants in this study underwent screening for eligibility criteria. Participants who met the following criteria were enrolled: (1) Experienced at least one fall in the six months before the study; (2) Ability to follow instructions and give written consent. Exclusion criteria included: (1) Visual or cognitive deficits (Mini-Mental State Examination score < 17) that interfere with operating a smartphone; (2) individuals who are pregnant or planning to be pregnant.

During their enrollment visit, we collected the participant’s medical history as well as the scores of the modified falls efficacy scale (MFES) which measures the participant’s confidence in performing ADLs without falling [40] and the World Health Organization’s Quality of life brief version (WHOQoL-Bref) which measures the participant’s quality of life in four domains: physical health, psychological, social relationships, and environment [41].

Each participant was given a Samsung Galaxy S5 smartphone running on Android 6.0.1 with a pre-installed sensor data collection app, called Purple Robot, and the custom fall-detection module enabled [42, 43]. Purple Robot is an Android application developed for modular collection and processing of data streams available on a smartphone device. It allows for collection and processing of hardware data signals, as well as customizable notifications based on the processed signal outputs. A custom fall-detection module, implementing a model developed as part of prior work, was developed for use in this study. Nationwide unlimited talk/text data plan was enabled on these phones. Participants were provided with a brief functional overview of the Purple Robot app and its key features on the smartphone. Participants received a charger and a waist pouch if they chose to carry the phone around their waist. They were instructed to carry the smartphone during their awake hours for a continuous period of 90 days. No lifestyle alterations were suggested, and participants were advised to carry out their day-to-day activities as usual. All participants were residents of the Greater Chicago Area, which includes the city and its suburbs, yet they were free to travel with the smartphone within the borders of the United States.

During the 90-day trial, on every occasion when the system detected a fall, it generated a notification on the participant’s smartphone and sent a text message to a member of the research team. Participants or designated caregivers were contacted by a member of the research team in the same or next business day after receiving a fall notification to verify the fall and gather information on their wellbeing. During this follow up call, the research member would also collect a narrative description of the participants’ activity at the time of the alert, including what triggered the fall and how long the participants remained on the ground after the fall (if applicable). Further, the participants were instructed to call the research team in any case they experience a fall but were not contacted by the team during the next business day. Additionally, a member of the research team initiated periodic check-in calls every 20 days and asked for any falls that occurred and were not reported.

Status of the deployed phones (battery level, data plan usage) was monitored by the research team using the web portal. Participants were contacted by a research team member in case of no activity on the phone for three consecutive days.

At the end of the 90-day trial, the participants were invited to a final visit in which they returned the smartphone and were asked to provide feedback during a semi structured interview on the experience of using the fall detection app. During this visit, the participants were once more questioned regarding any fall event that was not detected during their study participation.

### Fall detection model

**Fig. 1:**
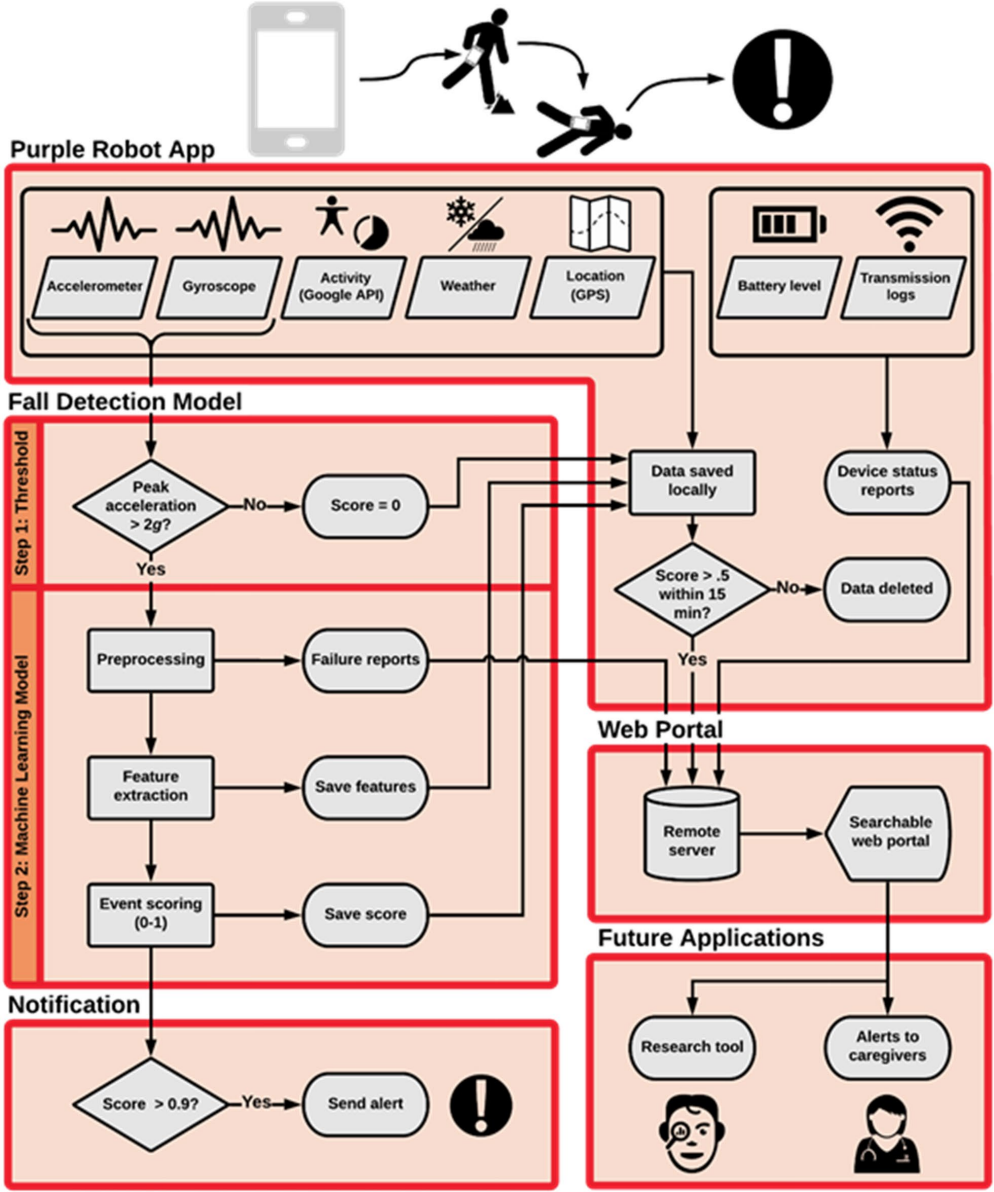
The fall detection system, including input data, fall detection model, system’s output, web portal, and future applications

We developed a hybrid model for the detection of real-life falls. The model includes a first-stage screening acceleration threshold and a second-stage machine learning classifier (see Fig. 1). The acceleration threshold screening reduces the computational burden by decreasing the number of events that had to be processed by the machine learning classifier.

This model was developed and validated using data from simulated falls and daily tasks performed in a laboratory setting by 17 participants [44]. The participants included seven individuals with transfemoral amputation and ten healthy young adults with no elevated fall risk. While simulating the falls, participants carried a Samsung S5 smartphone that recorded accelerometer and gyroscope data from the built-in hardware sensors using the Purple Robot application. During the simulated falls recording, a researcher used a second phone to log and label the timestamps of the events.

The recordings were then split into 5-second data windows, similar to the window sizes in previous studies [45–47]. In order to provide many different examples of falls occurring in different portions of the 5-second windows (i.e., beginning, middle, end of the window), for each fall event we created ten windows, each a randomly-selected 5-second range including the fall-impact. The fall-impact was identified as the maximum acceleration magnitude occurring within 2.5 s of the fall-timestamp labeled during data collection. The smartphone application has a limited ability to sample the accelerometer and gyroscope signal at a consistent rate, due to resource demands from other applications on the device. To account for sampling rate inconsistencies, the accelerometer and gyroscope signals within each five-second window were interpolated to 250 samples at intervals of 20 ms, corresponding to 50 Hz frequency. To avoid generating signal artifacts through the interpolation process, we discarded windows including less than 200 samples or having a gap between consecutive samples larger than 200 ms. No filtering was applied to the data. This procedure resulted in a total of 8452 five-second data windows (7874 corresponding to falls and 578 corresponding to non-falls), which were used to train to the fall detection model.

The first stage of the fall detection model was the acceleration threshold screening. The threshold value was determined by assessing the maximum acceleration amplitudes of simulated falls versus non-falls. We found that all simulated fall events had peak acceleration magnitudes larger than 2 g, while 83.9 % of non-fall windows had peak acceleration below this value. Thus, we selected 2 g as the threshold value to ensure maximum sensitivity to falls while screening out the majority of non-falls events. High sensitivity is desirable for a first-step data screening, so as to not incorrectly remove true fall events before the final model stage.

The second stage of the fall detection model included a machine learning classifier. The classifier was trained only with data windows (both fall and non-falls) that included a peak acceleration above 2 g (i.e., passed the first-stage screening). For each of these data windows, a set of 40 features were computed (20 for the accelerometer and 20 for the gyroscope signals; Table 1). These features were used as inputs to a logistic regression model, which output a posterior probability (0–1) of a fall given the sensor data. In the final implementation on the mobile phone, this was binarized to a value of 0 for a non-fall activity and 1 for a fall after application of detection threshold, which was the 5th percentile of posterior fall probabilities across all simulated falls in the training set. To remove redundant features and avoid overfitting, a regularization procedure was applied using Elasticnet with parameters α = 0.6 and λ = 0.015 which were found through a cross-validation hyperparameter tuning [48]. These hyperparameters were selected based on a parameter grid-search performed on leave-one-participant-out cross-validation folds. The hyperparameter tuning revealed consistent performance within each fold with respect to the grid-search range used. The hyperparameter values for the final model noted above were chosen because they representative of the center of the search space. The performance of this system was estimated using a leave-one-participant-out cross-validation, which resulted in an average area under the receiver operating characteristics (AUROC) of 0.995 (95-percent confidence interval (CI): 0.992–0.995). The decision threshold for the deployment model was set to 0.908, which is the fifth percentile fall probability among all simulated falls. This corresponds to an estimated sensitivity of 0.95 based on our simulated falls data. Applying the selected threshold resulted in the following average performance metrics: accuracy of 0.959 (CI 0.940–0.977), the sensitivity of 0.954 (CI 0.930–0.977), and specificity of 0.984 (CI 0.972–0.995). Training and analysis of the logistic regression model were performed in MATLAB 2017a. In this study we evaluated the model performance by measuring its precision (i.e., the fraction of true falls out of the detected falls), recall (the fraction of falls that were detected out of the falls that occurred), and F1-score (i.e., the harmonic mean of precision and recall).

**Table 1:** The features that were computed for each 5-seconds clip of the accelerometer and gyroscope data

Feature name mean	Number of features
Median	1
Standard deviation	1
Skewness	1
Kurtosis	1
IQR and derivative of IQR	2
Minimum and derivative of minimum	2
Maximum and derivative of maximum	2
Maximum, minimum, and IQR on each axis (x, y, z)	9

### Web portal and smartphone app

If the estimated fall probability value exceeded 0.908, the Purple Robot app generated two alerts: (1) A message appeared on the faller’s phone screen; (2) a text message sent to the phone of a member of the research team. In addition, the Google activity recognition application program interface (API) (30) was applied every 60 s to assess the type of activity being performed by the user in the preceding 60-s interval. To reduce the amount of data to be transmitted and to conserve battery life, records for the sensor signals, fall probabilities, and activity recognition data were only transmitted if they were included within 15 min of an estimated fall probability greater than 0.5. Regardless of the event fall probability, additional data recorded by the system included the Global Position System (GPS) coordinates and phone battery life, which was transmitted to the cloud server every 60 and 30 s, respectively. The Purple Robot application and custom fall detection model were programed in Java and compiled using Android Studio version 2.3.

In addition to the mobile application, a web portal was developed to allow the exploration of the collected data. The web portal enables the filtering of potential fall events by its probability. For each potential fall event (i.e., 5 s intervals with fall probability > 0.5) the portal presents the following details: (1) the fall date and time, based in the phone log; (2) the falling probability, based on the estimation of the fall-detection model; (3) the fall location, based on the GPS coordinates at the time of the fall; (4) the faller movement speed prior to the fall, based on average change in location over the five minutes before the fall; (5) the weather condition (i.e., sky conditions such as clear, cloudy, rainy) at the time and place of the fall, obtained from Weather Underground (https://www.wunderground.com), with the closest reported data point in both time and location and; (6) the faller’s activity at the time of the fall, based on the Google Activity Recognition API [49, 50], with the most likely activity and confidence level reported. The web portal includes additional fields of the type of fall (e.g., tripping forward, slipping backward), and fall confirmation, which was not implemented in the current study. The web portal was implemented using Django, which is an open-source Python framework for coding web services [51].

## Results

### Concept and prototype

**Fig. 2:**
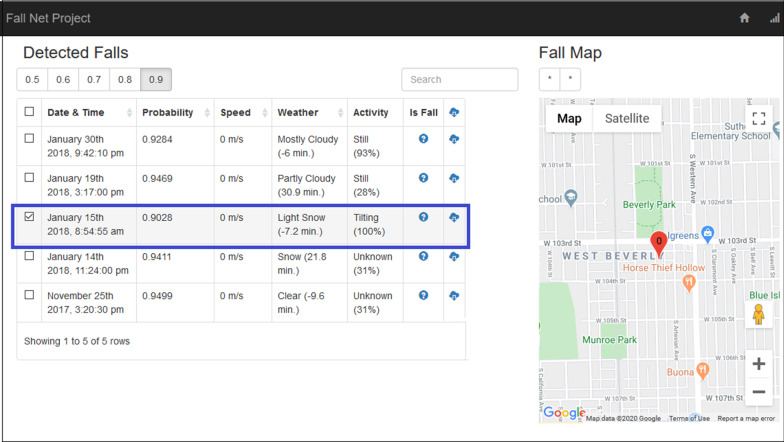
An example of the web portal data summary for a single study participant. Potential fall events are filtered by probability of being a fall, locations of events are visualized in the map, and metadata surrounding the event are displayed. The highlighted event presents the data of a real fall that was detected by the system

Our system uses the built-in hardware accelerometer and gyroscope sensors within a smartphone to monitor the user’s movements as they carry the phone throughout their day-to-day activities and detects when the user is falling (Fig. 1). All information regarding the fall (probability, location, activity recognition, weather) is assembled in a searchable web portal (Fig. 2), where researchers and clinicians can retrieve information about the fall characteristics and the faller status.

### Falls analysis—clinical data

We performed a prospective study in order to test the fall detection and response system for real-life falls in 23 individuals diagnosed as high risk of falling. The participant group included individuals who experienced lower-extremity amputation (n = 11), spinal cord injury (SCI; n = 5), stroke (n = 2), traumatic brain injury (TBI; n = 2), anti-SRP (Signal Recognition Particle) mediated polymyositis (SRP; n = 1), and Poliomyelitis (polio; n = 2). In this study, we defined a fall as “an unexpected event in which the participants comes to rest on the ground, floor, or lower-level’’ [52] and a stumble as “an event defined as a loss of balance regained before striking the ground” [28].

The demographic characteristics of the study’s participants are presented in Table 2. In total, each of the 23 participants (14 males) carried the phone for 90 days. The participants’ age ranged between 22 and 70 years, their height between 152 and 188 cm, and their weight between 51 and 142.9 kg. 32 % of the participants were obese (body mass index (BMI) > 30).

**Table 2:** Demographic characteristics of the participants

Characteristic	Mean	SD
Age (y)	48	13.7
Height (cm)	171.2	8.9
Weight (kg)	82.2	21.1
BMI (kg/m^2^)	28.1	7
Time since diagnosis (yrs.)	15	12
MFES Enrollment visit	8	1.6
Termination visit	8.2	1.5
WHOQOL (0-100)		
Physical health	68	18.5
Psychological	76.3	16.8
Social relationships	68.1	24.4
Environment	76.1	17.4

**Fig. 3:**
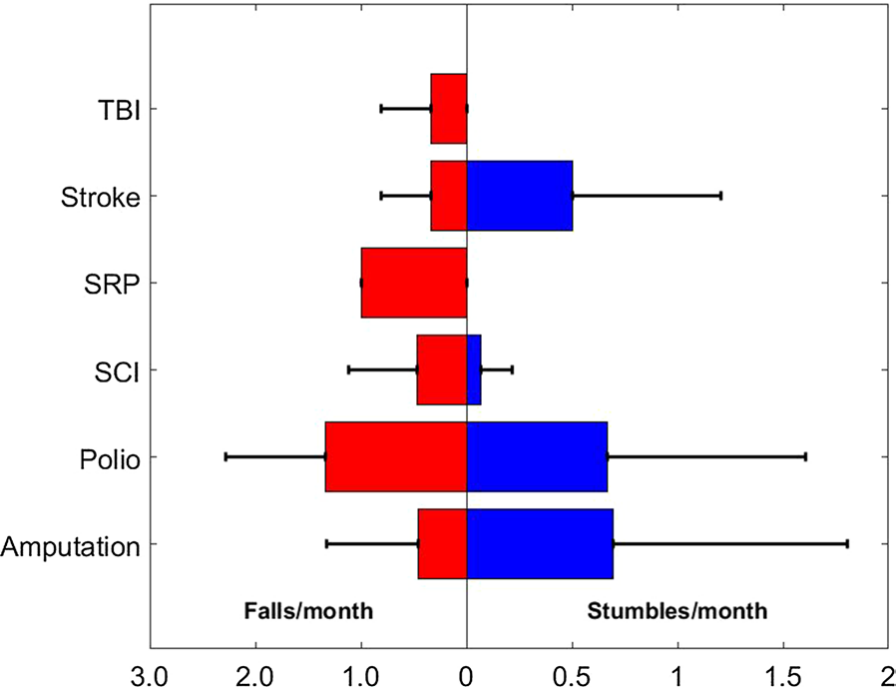
Average monthly rate of falls and stumbles for each condition. *TBI*  traumatic brain injury, *SRP * anti SRP mediated polymyositis, *SCI*  spinal cord injury, *Polio* poliomyelitis

During their 90-day trial, 13 participants (57 %) experienced at least one fall, and nine participants (39 %) experienced at least one stumble. These rates include both falls detected by our system and reported by the users. The monthly rate of falls and stumbles for each diagnosis group is presented in Fig. 3. This analysis demonstrates the potential of using the fall detection system as a potential tool to investigate the prevalence of falls and stumbles among different populations. The sample size of some of these groups was relatively small (see beginning of this clinical data section) and insufficient for performing population specific clinical inferences at this time.

**Fig. 4:**
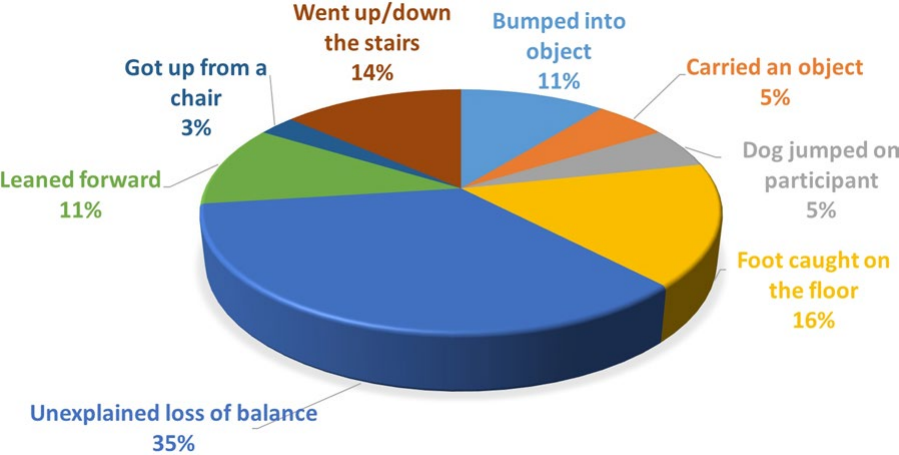
The activities performed before the fall, as reported by the faller

An analysis of the activity before the fall, as reported by the faller, is presented in Fig. 4. The results suggest that the leading causes for falling were impact with another object, which preceded 32 % of the falls (including foot caught on the floor, dog jumping on participant, and bumping into object), and changing the faller’s center of mass, which preceded 33 % of the falls (including leaning forward, getting up from a chair, carrying an object and going up/down the stairs). However, 35 % of the falls were reported to occur due to a sudden unexplained loss of balance. A faller’s report may be biased, especially as the time between the fall and the time to report increases [14]. Therefore, our fall detection system records a classification of the faller’s activity in the 60 s before the fall, currently based on Google’s activity recognition API. For the 27 true-falls that were detected by the phone, the system predicted the following activities before the falls: faller was standing still (26 % of the falls); tilting, where the angle relative to gravity changed significantly (41 %); on foot (11 %); bicycle (4 %); or unknown (18 %). Participants most often reported walking (49 %) or standing (30 %), including while reaching for an object or doing chores, at the time of a fall. Falling while in transition between two postures (e.g., sitting, standing, kneeling) was also reported (16 %). Much of the walking activity of participants was collected in the vague “tilting” category, though standing was generally correctly identified. Specific activities misclassified as “tilting” or “unknown” include everyday activities like getting out of a vehicle, carrying objects, reaching for household items, stairs assent/descent, shoe getting caught on carpet, knee buckling (prosthesis user) and getting up from wheelchair.

Our fall detection system records the weather condition at the time of the fall using Weather Underground. Such weather information can help to estimate the faller condition (e.g., during rainy or snowy days, a faller might require more assistance after a fall). Further, such data can help clinicians and researchers to understand the fall causation better. During the 2070 recording days, during 32.3 % of the total recording time the weather was clear, yet only 7 % of the falls occurred during clear weather. 57.6 % of the time the weather was cloudy yet 85 % of the falls occurred during cloudy weather. 4.2 % of the time the weather was rainy and 4 % of the falls occurred during rainy weather. 3.4 % of the time the weather was snowy and 4 % of the falls occurred during snowy weather. Since the system does not indicate if a fall was performed indoor or outdoor, the weather feature has only limited ability to contribute to the understanding of the fall activity.

### Fall detection model performance

An overview of the fall detection model’s performance metrics is presented in Table 3. During the 2070 days in which the system recorded participant’s activities, the phones analyzed 14,904,000 events (i.e., five seconds intervals of sensor signals). The participants reported 37 real falls, 27 of which were detected by the system (sensitivity = 73.0 %). Of the 10 falls not detected, six (60.0 %) did not exceed the 2 g acceleration threshold, either because the phone was not being carried at the time (confirmed for one case) or because the acceleration impact measured at the phone was low. The remaining four false negatives (40.0 %) exceeded the 2 g threshold but did not generate a high enough posterior probability from the logistic regression model to trigger an alert. In three of these four cases, participants reported falling to one knee rather than to a lying or sitting position; in the fourth case the participant was already kneeling at the time of the fall.

**Table 3:** Model performance metrics

Metric	Across all participants	Average (SD) per participant
Volume of recorded data (days)	2070	90 (0)
Sample size (5 s clips)	14,904,000	648,000 (0)
# Falls confirmed by participants	37	1.6 (2.25)
# Stumbles confirmed by participants	31	1.3 (2.55)
# Falls-related events detected by the phone	72	3.3 (3.72)
True positives (correctly classified falls)	27	1.2 (2.1)
True negatives (correctly classified non-falls)	14,903,928	647999.6 (0.89)
False positives (wrongly classified non-falls)	45	1.95 (2.65)
False negatives (wrongly classified falls)	10	0.43 (0.84)
Daily false alarm rate	0.5	0.02 (0.03)
Accuracy	99.9997 %	99.9996 % (0.0004 %)
Sensitivity	73 %	68 % (39 %)*
Specificity	99.9998 %	99.9997 % (0.0004 %)
Precision	37.5 %	41 % (41 %)**
F1-score	0.495	0.51 (0.33)*

*n = 13, only participants who experienced at least one fall, were included; **n = 16, only participants whose phone classified at least one event as a fall, were included

The system resulted in one false alarm per 46 days of use (Specificity > 99.9 %), falsely classifying a total of 45 additional events as falls (precision = 37.5 %). Out of these 45 events, 19 were confirmed by the participants to be stumbles, which are a risk factor for increased likelihood of falls and might result in injury [28]. Therefore, 63.9 % of all detections represented a fall or fall-like event, rather than a misclassification of an unrelated activity. Of the remaining 26 false positive events, 16 (62 %) were caused by the phone falling from unknown heights, two (8 %) involved transitions between sitting and standing positions, and one (4 %) occurred while in a vehicle. The remaining false positives (27 %) could not be attributed to a particular cause based on the participant interviews. When considering both precision and recall (sensitivity), our fall detection system resulted in overall performance (F1-score) of 0.495.

Only 45 samples out of the 14,904,000 that were assessed by the model failed to process (failure rate < 0.01 %). All failures but one were triggered by insufficient samples from the accelerometer, with the one remaining case having a sufficient number of samples but a time-lapse > 200ms without samples. The average battery life for the phones while actively running the fall detection system was 12.2 h (SD 5.0), meaning that the battery life was most likely not a restricting factor for collecting the data and detecting falls during regular daily activities.

## Discussion

We present a prospective study testing real-life falls detection using a smartphone-based online system. Our system detected 73.0 % of reported real-life falls with a relatively low false alarm rate (1 per 46 days of system use). This study shows the potential to use a smartphone-based system for detection of real-life falls in real-time. Due to the ubiquitous nature of smartphones, an app-based fall detection system would require no additional cost and very limited inconvenience (i.e., battery life, installation) for many users. In addition, our system utilizes the connectivity of the smartphone platform to enable timely notification of falls to a caregiver or emergency medical services. Reducing the time between a fall occurring and help arriving can help mitigate the impacts of falls ranging from head injuries, fractures, muscle damage, pneumonia, pressure sores, dehydration, hypothermia, and psychological consequences including increased fear of falling and related social isolation [7–11].

One challenge in developing a fall detection model with real-life falls data is the difficulty of recording real-life falls due to the rareness of these events [15, 16, 29]. An extensive collection of real-life falls is needed in order to develop detection models that perform well in practice. To collect such data and properly assess potential fall-detection models on new populations, a system capable of autonomously collecting, processing and filtering incoming data from many participants is essential. The system presented in this study can be readily adapted for use by a large number of individuals simply by a download into their smartphones—no additional hardware is needed. This system could serve as a research tool and scale the ability of future studies to capture data related to real-life falls.

Our system can periodically connect to a cloud server and update the parameters of its classification model. As a result, it can continuously learn from new data of real falls and improve the ability to detect future falls, though this was not explored in the current study and no model updates were made. The ability to continually learn and update the model may be especially useful, given the absence of a significant real-life falls database to be used for training the model. In the current study, the model resulted in different performances (i.e., sensitivity, precision, F1-score) for different diagnosis groups and different individuals (see clinical data section in the results for sample sizes for each group). While it should be noted that the sample size of some of the groups was relatively small, the performance difference may suggest the need to create a personalized, fall detection model to each user based on his/her individual-specific sensor data and fall recordings. Our system’s ability to continuously learn and update its detection model enables creating such personalized models.

**Fig. 5:**
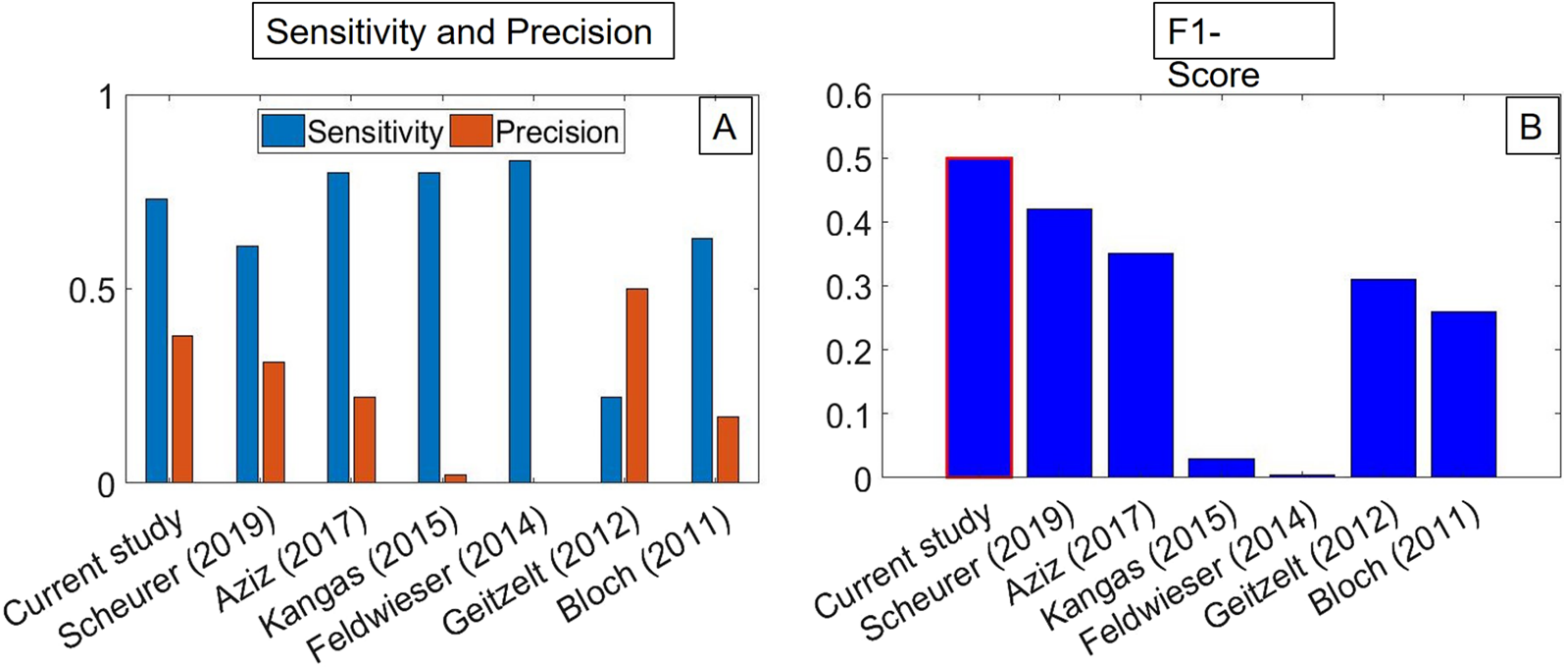
Comparison of real-life fall model performance metrics: **A** Sensitivity and precision; **B** F1-score

This study is the first prospective study that shows the ability to detect real-life falls in real time using a smartphone-based system. Nevertheless, it can be compared to previous prospective studies [30–36] which developed real-life fall systems using dedicated accelerometer sensors (Fig. 5). Only three systems [30, 31, 36] resulted in higher sensitivity (80.0-83.3 %) than our system (73.0 %). However, the precision scores in these studies (0.2–22.2 %) were lower than our system (37.5 %). Only one study [32], which employs accelerometers and optical sensors in a constrained home environment, resulted in higher precision than our system (50 %). However, the sensitivity of that system (22.2 %) was much lower than our system (73.0 %). Therefore, when considering both sensitivity (recall) and precision, using the F1-score, our fall detection system resulted in the highest performance (0.495). Even so, a direct comparison between the current study, which covered multiple causes of increased fall risk, and these previous works, which focused specifically on fall detection in the elderly, is only relative given the differences in sample populations. Despite our study focusing on a broad, rehabilitation-focused participant population, we find performance consistent with, and in places exceeding, prior studies focused on falls in elderly individuals. This does not guarantee the same performance when focusing only on elderly individuals, though.

Also, in comparison to previous prospective studies [30–36], the current study includes the largest number of recorded days (2070), and the largest number of events which were analyzed by the model (14,904,000). One of the desirable feature of our approach is scalability for large scale deployments to remotely and automatically upload the data from the phone to the cloud server as opposed to being manually downloaded from the sensors by a research staff member [31]. In addition, participants in the current study did not need to wear any special sensors and were not restricted to a constrained environment or any lifestyle adjustments to use this fall detection system. Though this study was still limited in terms of total number of participants compared to other large studies of fall detection [22], our approach has promise for future large-scale and long-term monitoring of at-risk populations.

Fall detection models can be divided into two groups based on their model approach: (1) a threshold-based model; (2) a machine learning model [18]. To our knowledge, out of prospective studies that used accelerometer data for detecting real falls, only the current study and Aziz et al. [30] used a machine learning approach (the current model is a mixed threshold and machine learning approach), while the rest [31–36] used a threshold-based approach. A comparison between the two groups reveals that on average, the machine learning model (including the current study) resulted in higher sensitivity (76.5 % vs. 61.7 %), precision (29.9 % vs. 19.9 %), and overall performance using the F1-score (0.421 vs. 0.204) than the threshold-based models. When comparing the machine learning models, our model was based on logistic regression, while the model of Aziz et al. [30] was based on an SVM (support vector machine) classifier. While the model of Aziz et al. resulted in higher sensitivity (80.0 % vs. 73.0 %), our model resulted in higher precision (37.5 % vs. 22.2 %) and higher overall performance using the F1-score (0.495 vs. 0.348). The tradeoff between precision and recall might lead to preferring one model over the other. In the case of fall detection higher recall (i.e., detecting more falls) might be more valuable than higher precision (i.e., detecting only falls). However, the optimal balance between the two should be investigated in future studies. In summary, the two prospective machine-learning classifier models performed comparably, and generally superior to the threshold-based strategies.

The increase in computational power available in recent years enabled several recent studies to apply deep learning method in fall detection models [23, 24, 53]. Yet, deep learning models tend to outperform other machine learning approaches in cases where large datasets are available. Fall detection datasets are relatively small (i.e., including only tens/hundreds of falls) which may be more suitable for a different machine learning model such as the regularized logistic regression applied in the current study. Further, while it is feasible to apply deep learning applications on a smartphone [54], continuously running such models (as required in a real-time fall detection system) might result in expensive computational cost [55]. The above reasons may partly explain why several smartphone-based applications in the field of translational medicine, including the current study, used less demanding machine learning methods [37–39].

## Limitations

The current study includes several limitations as follows. Since our system is smartphone-based, it is only relevant for individuals who have smartphones and are interested in carrying them. Further, the system will only be effective for the period in which the fallers carry the phone with them. Other phone-related variables such as hardware constraints impacting signal quality, battery life could influence the practicality of the proposed system. Battery capacity and performance could limit our potential to extend monitoring time beyond 12 h each day. Our smartphone-based system requires a minimum of 2G signal in order to send alert notifications and preferably 4G-LTE for exporting sensor data. Therefore, falls that will occur in locations without cellular reception will not be centrally detected for notification in real-time. Nevertheless, once the phone regains signal, the data is sent to the cloud server, and the fall is classified accordingly. The system does not indicate if a fall occurred indoor or outdoor which limits the ability to investigate the fall activity (e.g., the effect of the weather on the fall).

## Conclusions

In summary, we present a proof-of-concept fall detection system that can be implemented in a commodity smartphone to detect real-life falls and issue a timely notification to a caregiver or emergency medical services. The ubiquity of smartphones may increase the accessibility of a large global population to use this fall detection system. The system’s ability to automatically detect a fall in real-time, locate the faller, and send a notification could be critical for fallers that experience severe injury or remain unconscious, unable to call for help. The fall-related data stored by the system and available in the web portal may provide unique insights to be used by future studies to advance research on fall prevention, detection, and treatment.

## Data Availability

De-identified data are available from the authors upon reasonable request.

## Abbreviations

ADLs: Activities of daily living
CI: Confidence interval
GPS: Global positioning system
API: Application program interface
SVM: Support vector machine
2G: 2nd Generation
4G-LTE: 4th Generation-long term evolution
